# Drowning in Fluid: Post-infectious Glomerulonephritis Presenting as Acute Heart Failure

**DOI:** 10.7759/cureus.78753

**Published:** 2025-02-08

**Authors:** Stefan Gafoor, Walter Agyeman, Stanley Atencah, Christopher Chinnatambi, James Appiah-Pippim

**Affiliations:** 1 Graduate Medical Education, Piedmont Athens Regional Hospital, Athens, USA; 2 Internal Medicine, Piedmont Athens Regional Medical Center, Athens, USA; 3 Pulmonary and Critical Care Medicine, Piedmont Athens Regional Medical Center, Athens, USA

**Keywords:** acute kidney injury, decompensated heart failure, fluid overload, irgn, post-streptococcal glomerulonephritis

## Abstract

Staphylococcal infection is as common as streptococcal infection as a cause of infection-related glomerulonephritis (IRGN). It is seen more frequently in the pediatric population and is relatively rare in adults. Glomerular disease manifests as either nephritic or nephrotic syndrome. A high index of suspicion is necessary for diagnosing this condition as it can be mistaken for other common conditions in the elderly. IRGN may present as volume overload and may masquerade as cardiorenal syndrome type 1 in patients with preexisting heart failure, emphasizing the importance of distinguishing between the two because the treatment and prognosis may be different. We present an older adult male who was evaluated for suspected acute decompensation of heart failure in the setting of recent left knee septic arthritis and was found to have IRGN. This diagnosis may be delayed or missed due to it mimicking similar conditions and it requires a high index of suspicion.

## Introduction

Infection-related glomerulonephritis (IRGN) has been classily associated with *Streptococcus* species, but nonstreptococcal infections may cause it too. Staphylococcus infection has become a common cause of IRGN in the past 30 years, but it remains under-reported because the demographics and clinicopathologic features are ill-defined posing diagnostic challenges, especially in differentiating between the more common clinical conditions among the older aged population. The rise in post-Staphylococcus-related glomerulonephritis (PSRGN) cases may be attributed to drug-resistant *Staphylococcus* infections [1]. Our patient had a history of uncontrolled type 2 diabetes mellitus and recent septic arthritis complicated by methicillin-susceptible *Staphylococcus aureus* (MSSA) bacteremia. We suspect that immunologic dysfunction from his uncontrolled diabetes mellitus and vascular injury from MSSA bacteremia may have predisposed him to develop IRGN. The diagnosis can be made clinically in patients with nephritic syndrome who have an ongoing or preceding infection. Hypocomplementemia is common, and kidney biopsy may show proliferative glomerulonephritis on light microscopy and or hump-shaped subepithelial electron-dense deposits on electron microscopy. Other diagnostic tests may be useful, including serology and imaging such as renal ultrasound. The management focuses on antibiotic therapy of the underlying infection. It requires supportive treatment, but corticosteroids or cytotoxic agents may be used as adjuncts based on disease severity or disease progress despite adequate treatment of the causative organism.

## Case presentation

A 63-year-old male presented with a three-week history of dyspnea, two-pillow orthopnea, paroxysmal nocturnal dyspnea, and bilateral lower leg swelling up to his thighs. He had a history of hypertension, type 2 diabetes mellitus, and recent septic arthritis (three weeks ago). He had been admitted to the hospital three weeks prior for left MSSA prosthetic knee septic arthritis for which he underwent irrigation and debridement and was receiving IV ceftriaxone 2 g daily for six weeks. He weighed himself about four times a week and had noticed a progressive weight gain of 35 pounds over the past two weeks. He had an intermittent cough with white clear sputum production. He endorsed low-grade fevers and palpitations. He denied chest pain or calf pain. 

His medical history was significant for heart failure with improved ejection fraction, hypertension, urinary incontinence, and type 2 diabetes. His surgical history was notable for left knee arthroplasty. On initial presentation, he was afebrile, heart rate of 69, blood pressure of 169/95 mmHg, and oxygen saturation at 93% on room air. He was dyspneic at rest requiring 4 L of oxygen, with elevated JVP, bilateral crackles on posterior lung auscultation, and 3+ bilateral pitting lower extremity edema. 

His lab work was notable for hypochromic microcytic anemia with a hemoglobin of 12.1 g/dL (13.8-17.2 g/dL), elevated creatinine of 2.96 mg/dL (0.7-1.3 mg/dL) with his baseline 1.98 and albumin at 3 g/dL. His D-dimer was elevated to 3329 ng/mL (<500 ng/mL). Pulmonary embolism was ruled out with a normal ventilation/perfusion scan, making his recent infection with possible microvascular injury the most likely reason for his elevated D-dimer level. His brain natriuretic peptide level (BNP) was elevated at 439.4 pg/mL (<100 pg/mL) in the setting of his elevated BMI of 36.42 kilograms per meter squared; given this, his clinical presentation was most consistent with acute decompensated congestive heart failure. His urinalysis showed 2+ blood, 2+ proteinuria, negative nitrites, 13 red blood cells per high-power field (HPF) (0-4 per HPF), and 8 white blood cells per HPF (2-5 per HPF). Fractional excretion of urea was 19%, suggesting prerenal disease. 

ECHO showed an EF> 65%, moderate concentric LVH with moderate diastolic dysfunction, dilated IVC, elevated RVSP 50-60 mmHg, and a tricuspid regurgitant jet with Vmax of 3.14 m/s. Below is a table summarizing his laboratory workup and trend.

**Table 1:** Laboratory results of the index patient

Laboratory tests with reference ranges	06/19/22	06/13/22
White blood cell count (4-10.5 x10*3/uL)	5.20 x 10^3^/uL	5.70 x 10^3^/uL
Hemoglobin (13.8-17.2 g/dL)	10.1 g/dL	10.0 g/dL
Hematocrit (40-50.0 %)	30.7 %	30.4 %
Mean cell volume (78-100.0 fL)	74.1 fL	75.4 fL
Platelets (130-400 x 10*3/uL)	150 x 10*3/uL	230 x 10*3/uL
Sodium (134-144 mmol/L)	138 mmol/L	137 mmol/L
Potassium (3.5-5.2 mmol/L)	4.9 mmol/L	4.6 mmol/L
Chloride (96-106 mmol/L)	107 mmol/L	105 mmol/L
CO_2_ (20-29 mmol/L)	23 mmol/L	20 mmol/L
Blood urea nitrogen (6-20 mg/dL)	69 mg/dL	83 mg/dL
Creatinine (0.76-1.27 mg/dL)	1.98 mg/dL	2.96 mg/dL
Glucose (70-99 mg/dL)	246 mg/dL	157 mg/dL
Anion Gap (8-16) mmol/L	13 mmol/L	17 mmol/L
Calcium (8.4-10.2 mg/dL)	8.5 mg/dL	8.1 mg/dL
Aspartate aminotransferase (12-50 U/L)	20 U/L	25 U/L
Alanine transaminase (7-52 U/L)	21 U/L	27 U/L
Alkaline phosphatase 32-126 U/L)	137 U/L	158 U/L
Total bilirubin (0.2-1.4 mg/dL)	0.5 mg/dL	0.4 mg/dL
Albumin (3.0-5.0 g/dL)	3.4 g/dL	3.0 g/dL
Lactate (0.50-2.00 mmol/L)	0.6 mmol/L	-
D-dimer (<0.50 ug/mL)	3,329 ug/mL	-
Brain natriuretic peptide (10.0-100.0 pg/mL)	439.4 pg/mL	-

He responded well to intravenous diuresis with a urine output of 2 L net negative in 24 hours with improvement in respiratory symptoms. By day 4 of admission, his creatinine had improved with no residual dyspnea, and he was diuresed to a net negative 9.1 L; however, he continued to have bilateral lower leg edema. His initial acute kidney injury was thought to be from heart failure decompensation leaking to AKI (cardiorenal type I syndrome), which responded well with diuresis. However, the improvement in his creatinine stalled at 1.5 mg/dL despite adequate diuresis and urine output. Pivotal turning points that prompted further investigations into renal etiology for his AKI were persistent proteinuria, hematuria, and low albumin levels. The protein/creatinine ratio was elevated at 3200 mg/g (<150 mg/g) consistent with nephrotic range proteinuria. Possible etiologies included diabetic nephropathy, amyloidosis, and IRGN given his recent septic arthritis. Complement C3 and C4 levels were ordered that resulted in a C3 being low. Cardiac MRI and amyloid studies were ordered. Serum immunofixation showed no abnormal bands. His free kappa serum levels were elevated to 53.8 and free lambda levels were elevated to 29.4. His free kappa/lambda ratio was elevated at 1.83.

**Table 2:** Serologic and urinalysis results of the index patient

Test	Result	Normal range
C3 level	43 mg/dl	82-185 mg/dl
C4 level	42 mg/dl	15-53 mg/dl
Free kappa serum	29.4 mg/dl	3.3-19.4 mg/L
Free lambda serum	53.8 mg/L	5.7-26.3 mg/L
Free kappa/lambda ratio	1.83	0.26-1.65)
Protein/creatinine ratio	3200 mg/g	<30 mg/g
Urinalysis	Hazy, pH 5, Specific gravity –1.011, glucose=- 1+, ketones- negative, blood 2+, protein-2+, nitrites- negative, bilirubin- negative abd 13 RBC/HPF and WBC- 8/HPF. Urine eosinophils was 0.	
Serum immunofixation	No abnormal bands	
Autoimmune serology	ANA- negative, anti-GBM-negative, Anti-MPO antibodies, Anti proteinase 3 ab- negative	

**Figure 1:**
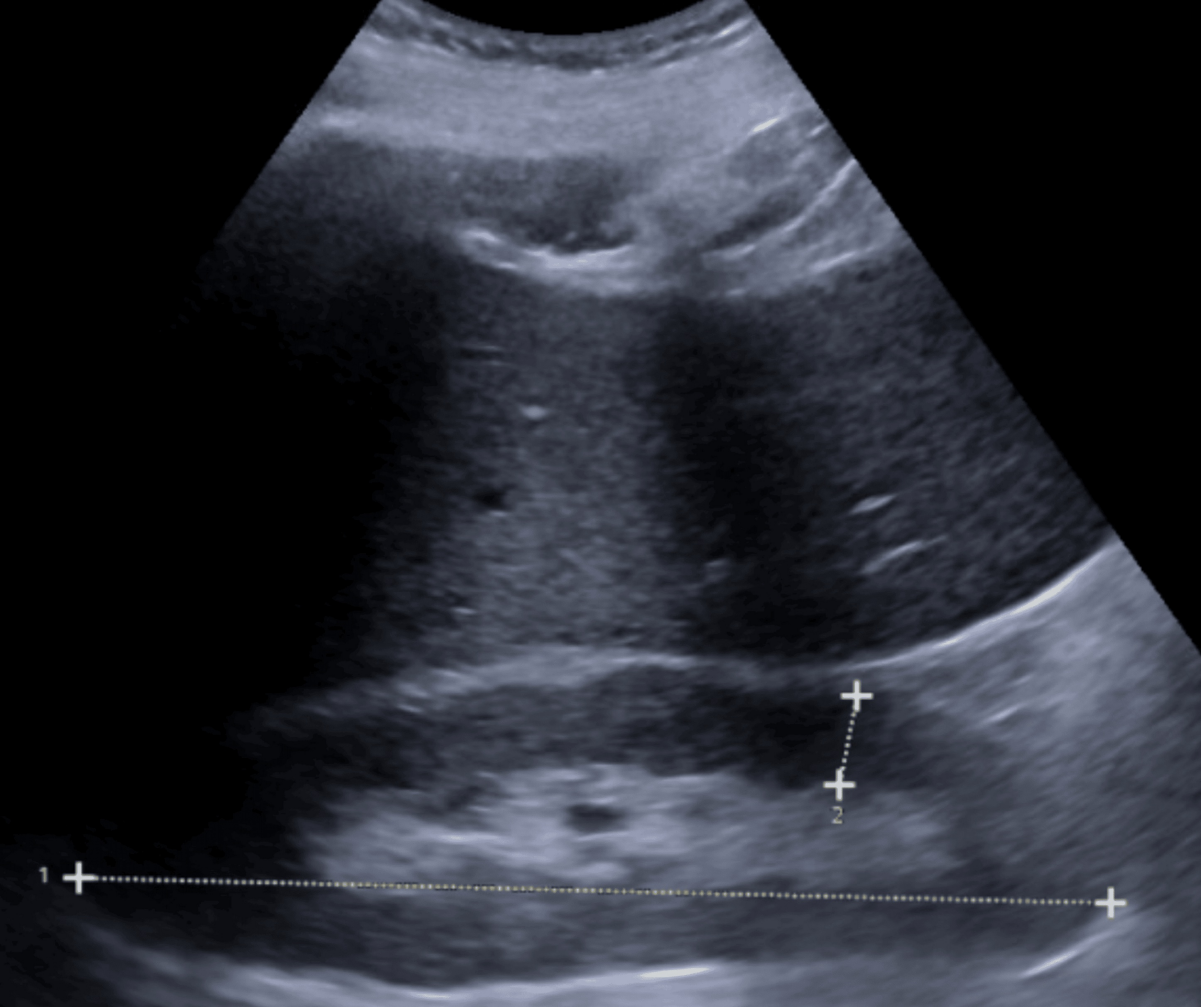
Renal ultrasound of the right kidney Line number 1 shows the right kidney measuring 15.67 cm in length (reference range: 10-14 cm). Line number 2 shows the right kidney cortical thickness of 1.38 cm (reference range: 0.7-1.1 cm).

His cardiac MRI showed poor nulling of the myocardium and mild concentric left ventricular hypertrophy, which were suggestive of amyloidosis. Renal ultrasound showed an enlarged right kidney measuring 15.7 cm and a left kidney measuring 12.5 cm without evidence of hydronephrosis (Figure 1). A non-obstructing left renal stone was incidentally noted. Bilateral anechoic renal cysts measuring up to 2.4 cm in the inferior pole right kidney and 9.5 cm in the upper pole left kidney were also noted. These findings were not in keeping with chronic renal disease as there were no renal parenchymal abnormalities nor were there any loss of cortical thickness that would usually be seen in chronic renal disease. These findings were however, non-specific and may be seen in the "hyperfiltration" phase of diabetic nephropathy, amyloidosis, or even IRGN. Given the asymmetry in kidney enlargement, we suspected diabetic nephropathy and amyloidosis to be less likely contributing factors as we would have expected enlargement in both kidneys. In our quest for a definitive diagnosis, a kidney biopsy was performed, which was stain-negative for amyloidosis, making this differential less likely. A proliferative and exudative immune complex-type glomerulonephritis was noted in a small cortical sample that was consistent with infection-related glomerulonephritis. All other autoimmune workups performed were unrevealing, including antinuclear antigen (ANA), anti-glomerular basement membrane (GBM), anti-myeloperoxidase (MPO) antibodies, and anti-proteinase 3 antibodies. After clinching the diagnosis, he was managed conservatively with the continuation of ceftriaxone for his MSSA infection, avoidance of nephrotoxic medications, and as-needed diuresis based on weight measurements and symptoms of dyspnea. Corticosteroids and cytotoxic medications were not administered due to his renal function stabilizing around a creatinine level of 1.5 mg/Dl with appropriate urine output.

## Discussion

The connection between the heart and kidney has been studied for over 200 years since Robert Bright described the prevalence of cardiovascular diseases in patients with renal disease distinguished by albuminuria in the year 1836 [2]. The National Heart, Lung, and Blood Institute defined cardiorenal syndrome as occurring because of interactions between the kidney and the circulatory compartment including the heart which worsens the symptoms of heart failure and the progression of heart and kidney disease [3]. Frequently, a cardiocentric approach is commonly observed where acute decompensated heart failure is majorly defined as the precipitating factor for cardiorenal syndrome.

In 2010, the Acute Dialysis Quality Initiative provided an update to the cardiorenal syndrome by grouping the condition into two main types, cardiorenal and renocardiac syndromes, based on the primary underlying cause of the disease process [4]. This led to the five subtypes based on the disease acuity and sequential organ movement (Table 3).

**Table 3:** Classification of CRS based on the consensus conference of the acute dialysis quality initiative CRS: cardiorenal syndrome, AKI: acute kidney injury, CKD: chronic kidney disease

Subtype	Name	Description
Type 1 CRS	Acute CRS	Acute heart failure resulting in AKI
Type 2 CRS	Chronic CRS	
Type 3 CRS	Acute renocardiac syndrome	AKI resulting in acute heart failure
Type 4 CRS	Chronic renocardiac syndrome	CKD resulting in chronic heart failure
Type 5 CRS	Secondary CRS	Systemic disease resulting in heart and kidney failure

While Occam's razor recommends that we seek the simplest explanation when faced with problem solving with more than one choice, Hickam's dictum by contrast states that many explanations are possible for a particular problem. The less apparent cause may be the culprit diagnosis. Our patient had four out of the five hallmark features of the nephritic syndrome, specifically edema, hematuria (with dysmorphic erythrocytes or erythrocyte casts), proteinuria, and hypertension, and kidney dysfunction may also occur. On the other hand, the classic features of the nephrotic syndrome include a urine protein excretion >3.5 g/24 hours or a urine protein-creatinine ratio >3500 mg/g, hypoalbuminemia, hyperlipidemia, and edema. He had a recent MSSA left knee arthritis with the removal of prosthesis, incision, and drainage and presented with an acute fluid-overloaded state with an AKI. The precipitating event for his clinical condition was initially believed to be heart failure decompensation with resultant AKI (cardiorenal syndrome type 1); later on in his hospitalization, we recognized that his AKI with fluid overloaded state was better explained by IRGN.

IRGN is an immune-mediated type II or III hypersensitivity reaction to infections occurring outside the kidneys but affecting the kidneys by causing nephritic syndrome - characterized by hematuria, nephropathy, hypertension, and reduced renal function. IRGN occurs more commonly in the pediatric population, two weeks after streptococcal pharyngitis and six after a bacterial skin infection. This is more commonly termed post-streptococcal glomerulonephritis (PSGN). However, over the last few years, there has been an interesting shift in the epidemiology, etiology, sites of infection, and prognosis across the globe [5]. Especially in the developed world, adults and particularly immunocompromised individuals are being diagnosed with IRGN at alarming rates, with prevalence reported at 0.3 per 100,000 person-years in developed countries and two per 100,000 person-years in developing countries [6]. The true prevalence may be higher as most patients have subclinical forms or do not undergo renal biopsies. The trend of diagnosis has decreased over the last three decades due to better living conditions and improved access to healthcare and antibiotics [7]. Nasr and colleagues in 2011 demonstrated significant differences between the causative organisms, sites of infection, and prognosis as compared to PSGN in children [8]. Adults, especially the elderly (>64 years), are increasingly implicated with a male predominance of 1.5-3.0 [9,10,11]. Caucasians are commonly affected compared to other racial backgrounds [8]. As seen in our patient, he was an older male with existing uncontrolled type 2 diabetes mellitus. 

Concerning bacterial etiology, this differs considerably from the usual PSGN. Non-streptococcal bacteria especially *Staphylococcus aureus* are incriminated in most IRGN. *Staphylococcus* infections are common in developed nations, while *Streptococcus* remains the leading cause in developing countries [8]. Diabetes mellitus, chronic alcoholism, and other chronic diseases like AIDS, tuberculosis, malignancy, and severe malnutrition remain significant risk factors for the development of IRGN [8,9]. In fact, diabetes mellitus was present in about half of adults diagnosed with IRGN in a study by Nasr and colleagues (2011) as in our patient [8]. The diabetes prevalence in IRGN could be higher with the increasing overall incidence across the world. Diabetes is a predisposition to Staphylococcal infections, likely due to *Staphylococcus aureus* skin colonization, which may be attributable to impaired immune function, increased skin and mucosal surface glycosylation, and altered skin barrier. Other non-streptococcal bacteria identified are *Staphylococcus epidermidis*, *Hemophilus*, *Pseudomonas,* and *Yersinia* [12,13]. Keller and colleagues also found chronic alcoholism to be an important risk factor, especially in the European adult population [11]. Sites of infections that are associated with IRGN are ubiquitous compared to just the upper respiratory tract and skin for PSGN. Aside from these common sites, infections in adults and diabetics tend to involve the urinary tract, lung, dental, heart, bones, joints especially prosthetic joints, and deep-seated visceral abscesses [8].

It has been postulated that bacterial antigens are nephritogenic, in that bacteria produce antigens that cause glomerular injury through several mechanisms. Antigens form complexes known as antigen-antibody immune complexes (AAIC) leading to kidney injury. The most commonly reported pathogenesis includes one of three ways. The first is antigen-antibody immune complexes that are passively entrapped in the glomeruli as mesangial or subendothelial deposits, and these AAIC cause activation of the classical and alternate complement pathways. The second mechanism includes the formation of glomerular AAIC directed against planted cationic bacterial antigens or intrinsic glomerular antigens via "molecular mimicry," leading to sub-epithelial deposits and activation of the alternative complement pathway. Lastly, the third mechanism involves bacterial antigens in glomeruli causing plasmin activation in the mesangium and glomerular basement membrane (GBM) leading to complement activation through the lectin or alternate pathways [5].

These mechanisms could occur in isolation or as a simultaneous process. This leads to the generation of C3a and C5a, which act as chemotaxins that recruit neutrophils and monocytes to cause glomerular injury by impairing mitochondrial function, antioxidant defense, and increasing cell motility. In addition, plasmin activation cleaves components of the glomerular basement membrane such as laminin, collagen, and fibronectin, which disrupts the integrity and function of the filtration barrier and induces chemokine and cytokines, which leads to the recruitment of neutrophils and macrophages, further exacerbating glomerular injury [5]. There is therefore a deficiency of complement during this inflammatory process [10,14]. The diagnosis of IRGN includes laboratory workup, but the final diagnosis is clinched by a renal biopsy. Typical renal biopsy findings include diffuse endocapillary proliferative and exudative glomerulonephritis, which is the most common presentation [9]. Other histologic findings include mesangial proliferative glomerulonephritis and focal endocapillary proliferative GN with membranoproliferative glomerulonephritis as the least common finding [9].

Clinical manifestations are similar to PSGN in children but differ in certain aspects. Nephritic syndrome characterized by new onset hematuria, proteinuria, AKI, hypertension, and peripheral edema are very common. The extent of proteinuria varies, with a reported range of 1-3 g/day in the study by Nasr and team (2013), with full-blown nephrotic-range proteinuria in approximately a third of the adult population [5]. Urinalysis is likely to show hematuria with or without red cell casts, with macroscopic hematuria reported in 20-50% of patients [10,14]. Elevated serum creatinine and acute kidney injury are common presentations. In the adult group, it may present as a new onset or exacerbation of heart failure, even in adults without co-existing cardiovascular risk factors. In 2011, Nasr and colleagues found that 25% of adults with IRGN presented with a new onset of exacerbation of existing heart failure due to the kidney’s inability to excrete water and salt [8]. Less common presentations include recurrent fever, purpuric skin rash, anemia, and hepatosplenomegaly [1]. There is a difference in prognosis for IRGN in adults compared to PSGN in children. Whereas most children and young adults will return to normal kidney function, adults with IGRN are more likely to have a poorer prognosis, especially in diabetics with existing glomerulosclerosis likely due to pre-existing glomerular injury, which makes these patients more vulnerable to further renal injury and delayed renal recovery. Adults present with severe AKI and about 50% will require dialysis for uremia [8]. In addition, almost half of adults will develop chronic kidney disease (CKD) and a third will develop end-stage renal disease (ESRD), with some undergoing renal transplants [5,10,14]. 

Our patient presented with signs and symptoms of decompensated heart failure in the setting of hypertension after a recent admission for left septic knee arthritis caused by MSSA. Diabetes mellitus may have put him at a higher risk for IRGN. His blood work revealed hypocomplementemia, and his urinalysis showed hematuria with proteinuria. Although he responded well to diuresis, his kidney function did not recover as expected for cardiorenal syndrome type I. His overall presentation was in keeping with cardiorenal syndrome type 3. Due to persistent acute kidney injury and persistent proteinuria, the patient had a renal biopsy, which confirmed the diagnosis of post-infectious glomerulonephritis, consistent with cardiorenal syndrome type III. Treatment should focus on the underlying infection; our patient received intravenous ceftriaxone for MSSA bacteremia from prosthetic joint infection. It requires supportive treatment, but corticosteroids or cytotoxic agents might have a role if disease progresses despite adequate antibiotic therapy of the causative organism.

## Conclusions

Non-streptococcal IRGN cases have been increasing over the past three decades, particularly in adults. It is often a diagnostic challenge because of the variability that exists in the demographic and clinicopathologic features of the condition. In children, IRGN usually presents acutely, typically following a streptococcal infection; however, in adults, IRGN usually presents insidiously, and atypical features may be present. Therefore, a high index of suspicion is warranted in making a timely diagnosis. During the diagnostic evaluation, it is important to keep in mind patient risk factors that are associated with an increased incidence of IRGN, such as older age, type 2 diabetes mellitus, and a history of preceding infection. Making the diagnosis is complex and includes a thorough workup with serum blood testing, urine studies, renal imaging, and biopsy. On renal biopsy, some pathognomonic histologic findings include global endocapillary hypercellularity, with neutrophils and subepithelial hump-like deposits. The key to treatment is the early identification of a causative organism to help guide targeted antibiotic therapy. Other potential treatment strategies include the use of systemic corticosteroids or cytotoxic agents, depending on the disease severity. Early intervention may prevent long-term kidney damage and potential long-term complications including chronic kidney disease or end-stage renal disease.
